# Enhanced Fatigue and Durability Properties of Natural Rubber Composites Reinforced with Carbon Nanotubes and Graphene Oxide

**DOI:** 10.3390/ma13245746

**Published:** 2020-12-16

**Authors:** Hao Guo, Peizhi Ji, István Zoltán Halász, Dávid Zoltán Pirityi, Tamás Bárány, Zongchao Xu, Long Zheng, Liqun Zhang, Li Liu, Shipeng Wen

**Affiliations:** 1 State Key Laboratory of Chemical Resource Engineering, Beijing University of Chemical Technology, Beijing 100029, China; 2018200559@mail.buct.edu.cn (H.G.); 2018210263@mail.buct.edu.cn (P.J.); 2020700020@mail.buct.edu.cn (Z.X.); 2017400089@mail.buct.edu.cn (L.Z.); zhanglq@mail.buct.edu.cn (L.Z.); 2 Beijing Engineering Research Center of Advanced Elastomers, Beijing University of Chemical Technology, Beijing 100029, China; 3 Department of Polymer Engineering, Faculty of Mechanical Engineering, Budapest University of Technology and Economics, Műegyetem rkp. 3., H-1111 Budapest, Hungary; halaszi@pt.bme.hu (I.Z.H.); pirityid@pt.bme.hu (D.Z.P.); barany@pt.bme.hu (T.B.)

**Keywords:** natural rubber, graphene oxide, carbon nanotubes, crack precursor, fatigue properties

## Abstract

Fibrous carbon nanotubes (CNTs) and lamellar graphene oxide (GO) exhibit significant advantages for improving the fatigue properties of rubber composites. In this work, the synergistic effect of CNTs and GO on the modification of the microstructure and fatigue properties of natural rubber (NR) was comprehensively investigated. Results showed that CNTs and GO were interspersed, and they formed a strong filler network in the NR matrix. Compared with those of CNT/NR and GO/NR composites, the CNT-GO/NR composites showed the smallest crack precursor sizes, the lowest crack growth rates, more branching and deflections, and the longest fatigue life.

## 1. Introduction

Due to its excellent elasticity and viscoelasticity, natural rubber (NR) is widely used in the manufacturing of various products such as tires, hoses, conveyor belts, seals, and shock-absorbing and damping components [1]. Various rubber products operate under acidic, alkaline, oily, high-load, or high-temperature conditions [2,3,4,5,6]. Under long-term cyclic loadings, rubber materials gradually generate microcracks and eventually break, which can significantly deteriorate their mechanical properties. Therefore, new methods to improve the fatigue resistance of rubber materials and accurately predict their fatigue life are always necessary.

The type, content, and dispersion of nanofillers have a significant influence on the microstructural, static, mechanical, and dynamic fatigue properties of rubber composites [7,8]. Crack propagation is highly related to the type of the filler network. Conventionally, more than 60 phr of spherical fillers, such as: carbon black and silica, are added into the rubber matrix to improve its fatigue properties. However, the addition of a large amount of fillers can lead to their poor dispersion in the rubber matrix. Agglomerates with larger particle sizes cause local stress concentration [9,10,11,12], which can lead to the formation of microcracks at an earlier stage of the rubber fatigue process [13,14].

Graphene (GE) comprise large lamellar structures [15,16] and carbon nanotubes (CNTs) exhibit high aspect ratios [17,18]; therefore, they have been extensively studied as fillers in rubber composites. Dong et al. [19] and Xu et al. [20] introduced GE or CNTs as fillers to the rubber matrix; they discovered that the addition of even a small amount of GE or CNTs tend to significantly improve the electrical [21] and thermal conductivity [22], air tightness [23], and fatigue properties of the rubber composites. However, large GE agglomerates were easily formed due to Van der Waals forces between GE sheets. The fine dispersion and alignment of GE sheets in the polymer matrix were hardly achieved [24]. There are many functional groups on the surface of GO, which makes it easy to disperse in water. The GO/rubber composites could be obtained by mixing the GO solution with rubber latex, and the following cocoagulation [25]. Therefore, the GO dispersion and the interaction with rubber macromolecules were both greatly improved, resulting in enhancing the mechanical properties. In addition, embedding inorganic nanomaterials into the polymer composite could provide special properties. For example, CNTs were introduced into the polymeric materials and the prepared composites could be used for carbon dioxide separation at humidified conditions [26]. Polymers of intrinsic microporosity blending with nanomaterials showed antiphysical aging properties [27].

In our previous research [28], GO and CNTs were compound with carbon black filled rubber, respectively. The filler networks by formed by GO or CNTs with spherical carbon black were enhanced, due to the large aspect ratios of GO or CNTs. The enhanced filler network withstood a large stress, consumed more energy, and resisted crack propagation. Inspired by the complicated filler network, CNTs and GO with large aspect ratios were both introduced into the NR matrix to explore the effect of CNTs-GO network on the fatigue performance of the NR composites in this research. In order to ensure the fine dispersion of GO, an aqueous mixture of GO and NR latex was compounded using flocculation to maintain the fine dispersion of GO in the mixture. Thereafter, the CNT bundles, which easily disperse into a rubber matrix, were blended with the GO/NR composites to obtain the final CNT-GO/NR composites. The filler dispersion, CNT-GO filler network, mechanical properties, and strain-induced crystallization of the CNT-GO/NR composites were studied. Furthermore, the fatigue properties were thoroughly investigated, including the uniaxial fatigue life, crack precursor size, crack growth rate, and crack tip propagation path.

## 2. Experimental

### 2.1. Materials

NR latex was supplied by the Shanghai Yitai International Trade Co., Ltd. (Shanghai, China); CNT bundles were obtained from Zhenjiang Tian Nai Technology Co., Ltd. (Zhenjiang, China). GO was prepared according to the modified Hummers method [29,30,31,32,33]. All the other materials were commercial reagents.

### 2.2. Materials Preparation

Mechanical mixing is the traditional method for preparing rubber nanocomposites. However, GO sheets easily aggregate due to the strong Van der Waals forces between them. Incorporation of GO sheets directly into NR via mechanical mixing can result in the poor dispersion of GO. Therefore, the preparation of the GO/NR composites in this research includes three steps. (1) Preparation of an aqueous mixture of GO (2 mg/mL) by sonication (800 W) at 50 °C for 1 h and (2) addition of a calculated amount of the GO suspension into the NR latex under intense stirring for 30 min. The GO/NR mixture was then cocoagulated using a 1.0 wt % calcium chloride solution to obtain the GO/NR masterbatch. The resulting masterbatch was washed with deionized water five times and dried in an oven at 50 °C for 24 h, (3) the other ingredients were filled into the GO/NR masterbatch via mechanical blending to obtain the GO/NR composites.

CNTs easily entangle with each other due to their large aspect ratios. Therefore, CNT bundles with lengths of up to 50 µm and diameters between 6 and 8 nm were chosen as they easily dispersed into the rubber composites via mechanical blending [21,22,23]. The CNT bundles were added into the GO/NR masterbatch to obtain the CNT-GO/NR composite via mechanical blending. For comparison, GO/NR composite was prepared by latex compounding, whereas the CNT/NR composite was prepared by mechanical blending. The compositions of the different samples are shown in Table 1.

### 2.3. Characterization

The dispersion of the fillers in the NR matrix was observed using scanning electron microscopy (SEM, S4800, Hitachi, Tokyo, Japan) and transmission electron microscopy (TEM, Tecnai G220, FEI, Hillsboro, OR, USA). The filler network was investigated using a rubber process analyzer (RPA2000, Alpha Technology, Hudson, OH, USA) at a frequency of 1 Hz and 60 °C for a strain range of 0.28%–400%. The dynamic mechanical properties of the composites were tested using a dynamic mechanical analyzer (VA3000, Metra VIB, Limonest, France) under tension using a frequency of 1 Hz in the range of −80–80 °C at a heating rate of 3 °C/min. The mechanical properties were measured using an electronic tensile machine (CMT4104, Xin Sansi, China) according to the ISO 37: 2011 and ISO 34-1: 2010 standards.

The modified Mooney–Rivlin model [34] was used to analyze the strain-induced crystallization (SIC) of the NR composites, as shown in Equations (1) and (2):

(1) $$\sigma =2\left(C_{1}+\frac{C_{2}}{\lambda }\right)F(\lambda )$$

(2) $$F(\lambda )≅\left(1+\frac{\lambda^{2}}{3\lambda_{m}^{2}}\right)\left(\lambda -\frac{1}{\lambda^{2}}\right)$$

where *σ* is the nominal stress, *C*_1_ and *C*_2_ are the Mooney–Rivlin constants, *λ* is the stretch ratio, and *λ_m_* is the maximum stretch ratio when the specimen breaks. A linear equation was obtained by fitting the curves of *σ*/*F (λ)* and *λ*^−1^, and the values of *C*_1_ (intercept) and *C*_2_ (slope) were calculated from the linear equation. *λ_up_* indicates the point of the SIC, and was determined using Equation (3).

(3) $$\lambda_{up}^{3}=\frac{3\lambda_{m}^{2}C_{2}}{2C_{1}}$$

The fatigue lives of the NR composites under different strains (100–300%) were obtained using a fatigue testing machine (FT3000-2, De Mesia, Beijing, China), according to the ISO 6943:2007 standard.

The sizes of the crack precursors were determined using the critical tearing energy method, as described in our previous report [35]. The crack growth rates of the composites were determined using a crack extension analyzer (DMA + 1000, METRA VIB, Lyon, France) at 20 Hz and 25 °C. The dimensions of the samples were 2 mm × 6 mm × 40 mm, and the precut depth was 1.5 mm [36]. Under pure shear conditions, the tearing energy of the rubber composites can be calculated using the following equation:

(4) $$G=\frac{E_{f}}{S}=\frac{E_{f}}{L\times e}$$

where *G* is the tearing energy (J/m^2^), *E*_f_ is the input energy (J), *S* is the cross-sectional area of the sample (m^2^), and *L* and *e* are the width (m) and thickness (m) of the sample, respectively.

## 3. Results and Discussion

### 3.1. Filler Dispersion

**Figure 1:**
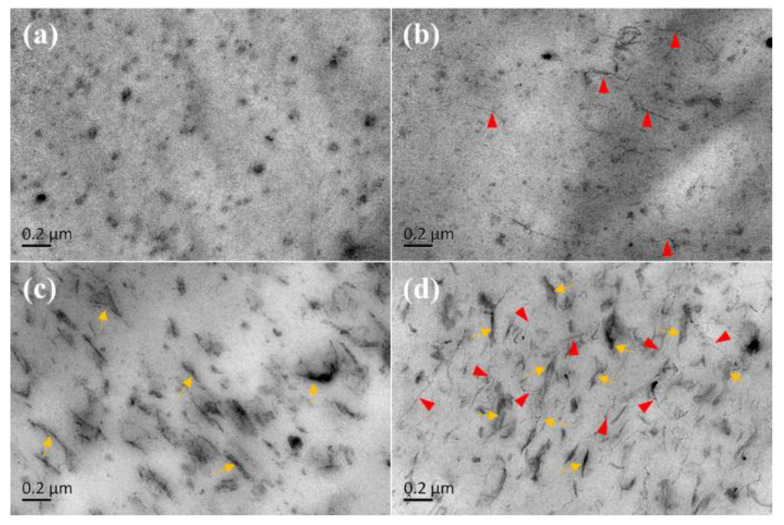
TEM images of natural rubber (NR) composites (**a**) NR, (**b**) CNT/NR, (**c**) GO/NR, and (**d**) CNT-GO/NR.

In order to clearly observe the dispersion state of CNTs and GO in the NR matrix, TEM images of the different NR composites were obtained, as shown in Figure 1. CNTs and GO sheets are marked by the red arrows and yellow arrows, respectively. The unfilled NR matrix in Figure 1a displays various black spots, which were identified ZnO particles [37]. Figure 1b shows linear CNTs that were evenly dispersed in the NR matrix, owing to the CNT bundles that were used. Figure 1c shows that the GO sheets were evenly dispersed in the NR matrix, originating from the fine dispersion of GO in the aqueous mixture and the subsequent flocculation [20]. Figure 1d shows that GO and CNTs were interspersed with each other, in a fishing net-like structure.

### 3.2. Filler Network

**Figure 2:**
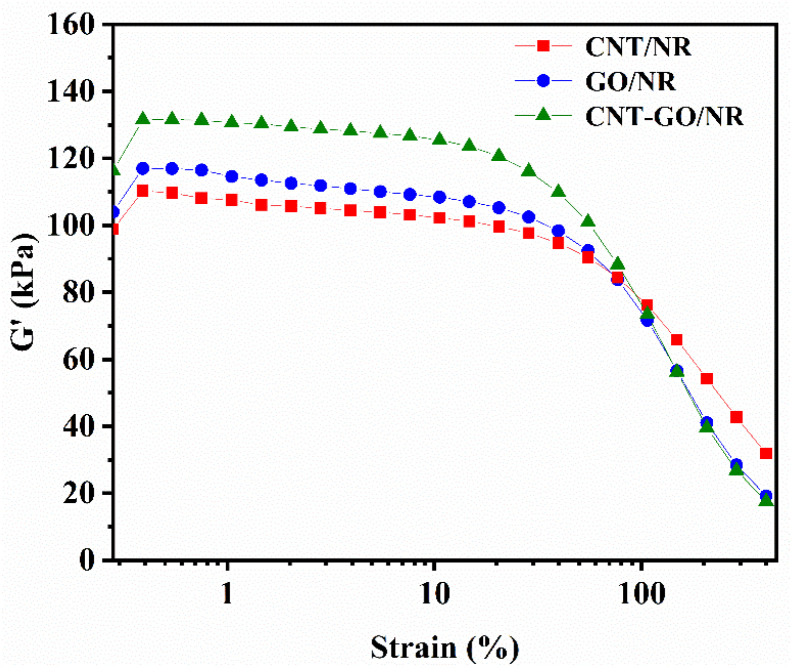
Curves of storage modulus (G′) versus strain of NR composites.

The curves of the dynamic storage modulus G′ and strain are shown in Figure 2. As the shear strain increased, the value of the storage modulus decreased due to the gradually increasing damage in the rubber network structures. When the strain reached the threshold, the initial storage modulus of NR dropped sharply, exhibiting a typical “Payne” effect. The main intermolecular interaction between the fillers are Van der Waals forces, which were limited by the distance between the fillers Under the same filler content, the storage modulus of the CNT-GO/NR composite was higher than that of the GO/NR and CNT/NR composites, indicating that the CNT-GO/NR composite has the strongest filler network among the composites. Under the same strain, the CNT-GO/NR composite resisted more force and stored more energy. To analyze the dispersion of the fillers, the absolute value of ∆G′ under 50% strain was used. As shown in Table 2, the ∆G′ of CNT-GO/NR was higher than that of GO/NR and CNT/NR, indicating that the interspersion of CNT and GO was beneficial to the dispersion of the CNT and GO fillers in the NR matrix.

### 3.3. Dynamic Mechanical Analysis

**Figure 3:**
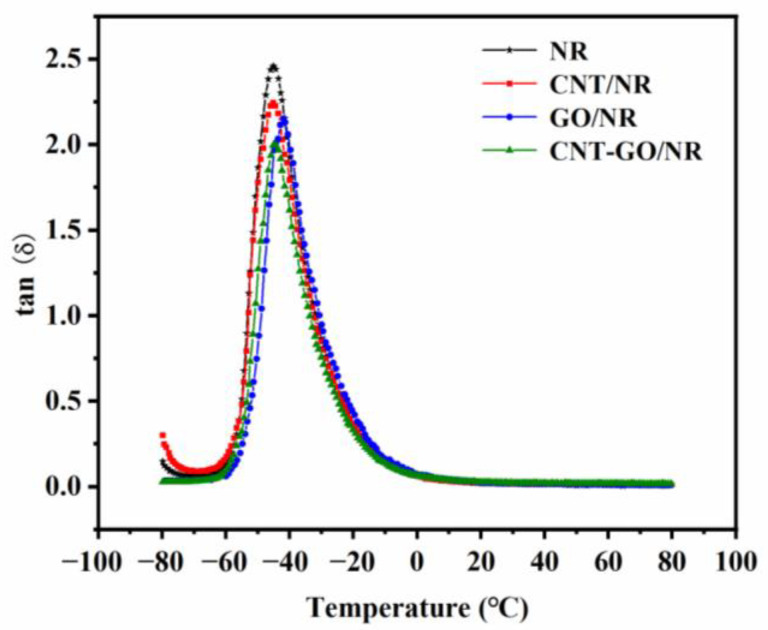
Curves of loss factor (tan δ) versus temperature of NR composites.

The dynamic mechanical properties of the NR composites are shown in Figure 3. Among the four samples, the unfilled NR had the highest loss factor. This is because the macromolecules of the unfilled NR moved violently once the matrix reached the glass-transition temperature. The friction between the rubber molecular chains caused very large energy losses and produced a large amount of heat. After the addition of CNTs or GO, the loss factor of the NR composite decreased because CNTs and GO restricted the movement of the molecular chains. Under the same filler content, the loss factor of CNT-GO/NR was lower than that of GO/NR. This is because the fishing net-like CNT-GO network structure tended to more strongly restrict the movement of the rubber molecular chains as compared to the restriction provided by GO sheets.

### 3.4. Mechanical Properties

The mechanical properties of the NR composites are shown in Table 3. Compared with those of pure NR, the NR composite with 1 phr of CNTs exhibited a higher tensile strength, superior elongation at break, larger 100% modulus, greater hardness, and better tear strength, thereby indicating that CNTs provided an effective reinforcement in the NR composites. Interestingly, with the same filler content, the tensile strength, 100% modulus, and tear strength of the CNT-GO/NR composite were higher than those of the GO/NR composite. This improvement indicates that the filler networks formed by GO and CNTs were significantly stronger than that formed by the GO sheets only, which was further confirmed by the RPA test in Figure 2. For further significantly increasing the mechanical properties of CNT-GO/NR composite, more GO and CNT contents are needed to form a strong filler network and absorb more NR macromolecules around nanofillers.

### 3.5. Strain-Induced Crystallization

**Figure 4:**
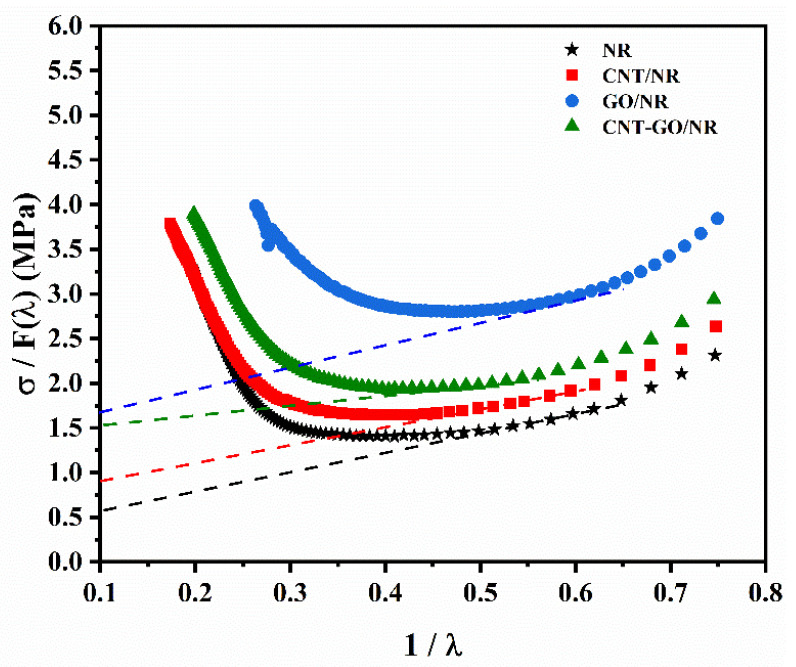
Modified Mooney–Rivlin plots for the NR composites.

The SIC performance of NR composites has a significant effect on their crack propagation rate. Therefore, the modified Mooney–Rivlin model was used to analyze the effect of GO on the SIC performance of the NR composites. The curves of σ/F(*λ*) and *λ*^−1^ are shown in Figure 4, and the values of *C*_1_, *C*_2_, and *λ_up_* are listed in Table 4. The value of *C*_1_ is indicative of the cross-linking density and elastic modulus of the NR composites The CNT-GO/NR composite had the highest cross-linking density among the four samples, and its *C*_1_ value was also the highest. Compared with those of pure NR, the CNT/NR composites had a lower *λ_up_* value, thereby indicating that the point of occurrence of the SIC in CNT/NR appeared earlier than that in pure NR. The *λ_up_* value of the CNT-GO/NR composite was even lower than that of the CNT/NR composite, signifying that the point of occurrence of the SIC appeared even earlier; this indicates that the SIC performance of the CNT-GO/NR composites was further improved by the addition of GO. The main reasons for the improvement of the SIC performance are as follows. First, the addition of GO sheets and CNT bundles led to the formation of more chemical cross-links and physical entanglement points in the NR composites. Second, the fibrous CNTs and sheet-like GO were arranged and oriented during stretching. Further, the conformational entropy of the rubber molecular chain became smaller, rendering it easier for the rubber molecular chains to orient and crystallize, thereby increasing the crystallinity of NR.

### 3.6. Fatigue Performance

#### 3.6.1. Fatigue Life

**Figure 5:**
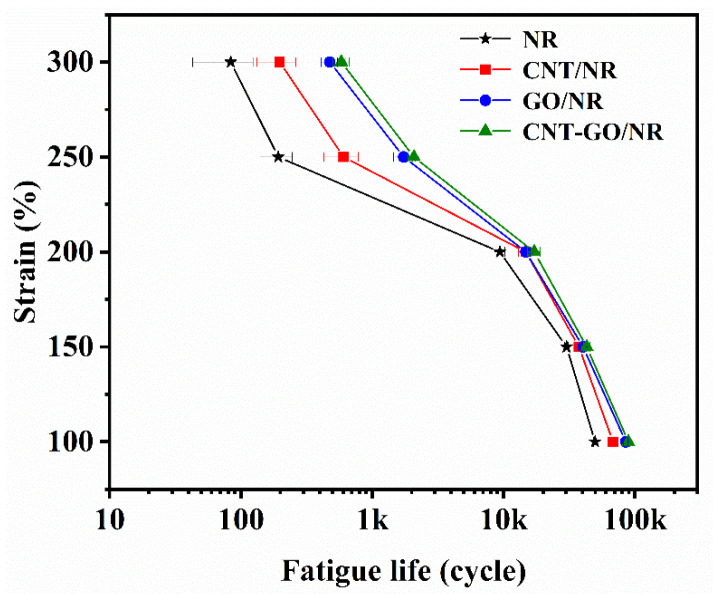
Fatigue lives of the NR composites under different strains.

S–N curves are often used to describe the relationship between fatigue life (N) and damage factor (S). The loading conditions of rubber are generally controlled by displacement during the tests and are easy to measure; hence, strain was used as the damage factor to predict the fatigue life of the rubber composite in this research. The S–N curves of the different NR composites are shown in Figure 5 The fatigue lives of the NR composites shown in Table S1. decreased as strain increased; this is because the rubber molecular chains rapidly broke down due to the application of a large external force during the cyclic stretching process at large values of strain. Under the same strain, the CNT-GO/NR composite had the longest fatigue life among the four samples. The main reason for this result is that the filler network in the NR matrix gradually improved with the addition of CNTs and GO, which effectively resisted large amounts of external stress. In addition, CNTs and GO improved the SIC performance of the NR composites, which was critical to fatigue resistance. With the same filler content, the CNT-GO/NR composite had a longer fatigue life as compared to those of the GO/NR composites, thereby indicating that the synergy between CNT and GO positively affected the fatigue life of the NR composites.

#### 3.6.2. Crack Precursor Size

Some defects such as the crack precursor (*c*_0_) are inevitably introduced into the rubber matrix during processing [38]. The sizes of the crack precursors usually range from 0.01 to 0.1 mm [14]. These crack precursors gradually expand into macrocracks under cyclic loadings; and eventually lead to fracture of rubber composites. Therefore, the size of the crack precursor is an important factor that affects the mechanical properties and fatigue lives of rubber composites [39].

The average crack precursor sizes of the different NR composites are shown in Table 5. The average crack precursor sizes of NR with CNT (1 phr) and GO (2 phr) were both smaller than that of the unfilled NR, thereby indicating that CNT and GO contributed to the reduction in the size of the crack precursor and were beneficial for improving the tensile strength and the elongation at break of the NR composite. With the same filler content, the average size of the crack precursor in the CNT-GO/NR composite was smaller than that of the crack precursor in the GO/NR composite, and the mechanical properties of the former were better, these results indicated that the synergy between CNT and GO enhanced the filler network and reduced the size of the crack precursor in the NR composites.

**Figure 6:**
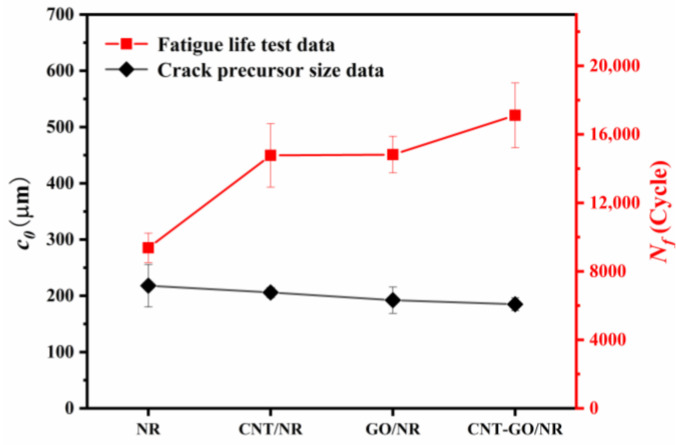
Crack precursor sizes and fatigue lives of different NR composites.

As shown in Figure 6, the crack precursor size was closely related to the fatigue life of the NR composites. The NR composite with a small crack precursor size had a long fatigue life, due to the following reasons: first, the GO and CNT introduced into the NR matrix formed a complex and strong filler network; and reduced the size of the initial defects of NR, thereby prolonging the duration of crack nucleation; second, the special structures of CNT-GO were conducive for enhancing the SIC performance of NR, which was beneficial for hindering crack growth and reducing the crack growth rate, thereby prolonging the fatigue life.

#### 3.6.3. Crack Propagation Rate

**Figure 7:**
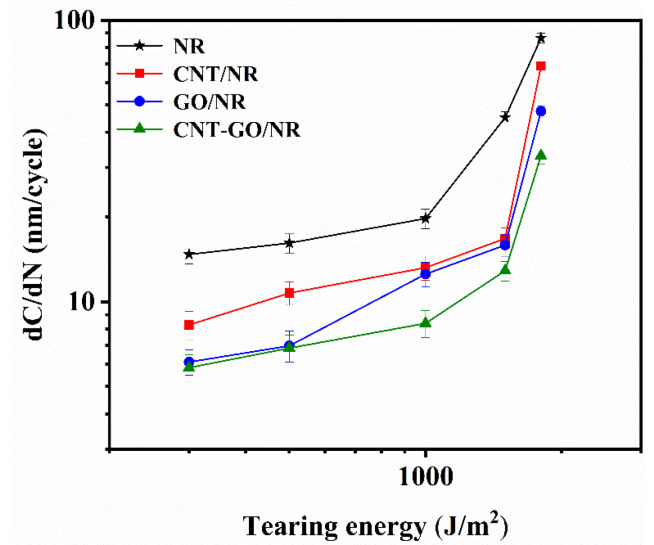
Crack propagation rate of NR composites under different tearing energies.

The crack growth rates of the NR composites under different tearing energies are shown in Figure 7 and Table S2. The crack growth rates of all NR composites increased with the increase in tearing energy. At low tearing energies (<500 J/m^2^), the crack growth rate did not change significantly, while at high tearing energies (>1000 J/m^2^), the crack growth rate increased exponentially. The crack growth rate of CNT/NR was significantly lower than that of the unfilled NR. When the tearing energy was 1800 J/m^2^, the crack growth rate reduced by 20.6%, indicating that the introduction of the fibrous CNTs was beneficial in reducing the crack growth rate of NR composites. Under the same tearing energy, the crack growth rate of CNT-GO/NR was lower than that of GO/NR, owing to the more complex network of CNT-GO as compared to the single GO network. The hysteresis energy of CNT-GO/NR was larger than that of the GO/NR composites, thereby leading to a higher reduction in the crack growth rate in the former. More importantly, the CNT and GO in the crack tips became oriented during the stretching process, which improved the SIC performance of NR and ultimately reduced the crack propagation rate of the composite.

#### 3.6.4. Crack Tip Evolutions

**Figure 8:**
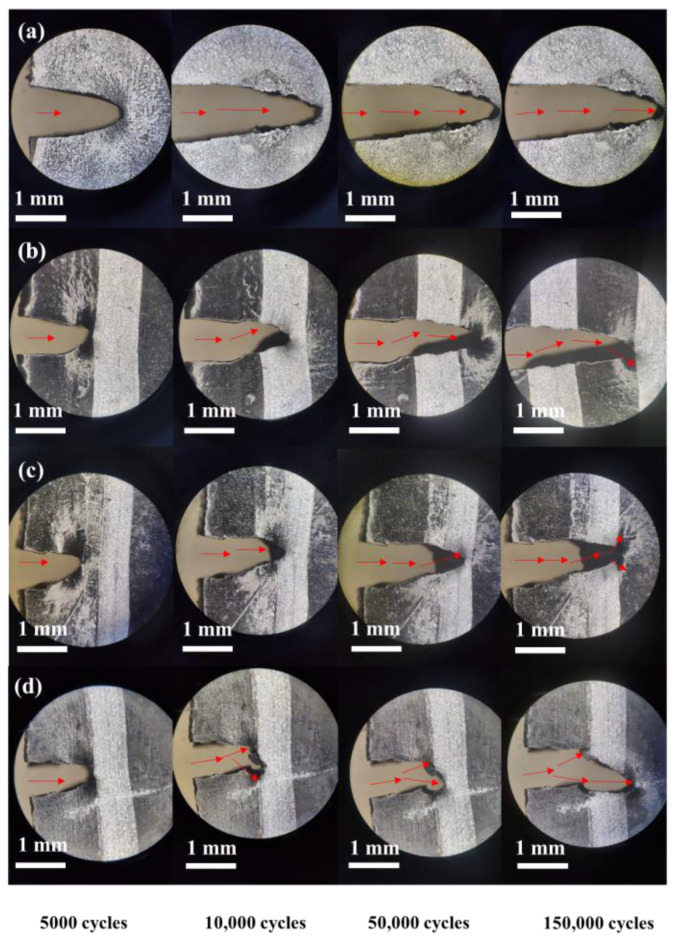
Crack propagation paths of different NR composites at 1000 J/m^2^ (**a**) NR, (**b**) CNT/NR, (**c**) GO/NR, and (**d**) CNT-GO/NR.

**Figure 9:**
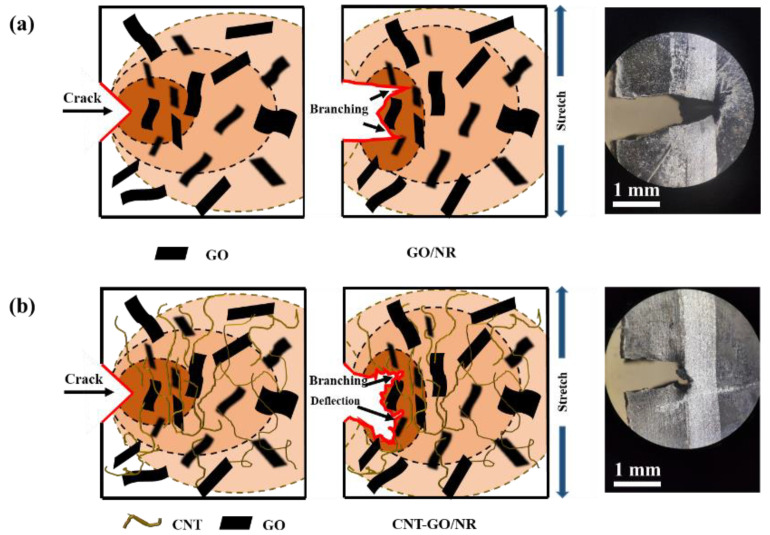
Schematic of the crack propagation in (**a**) GO/NR and (**b**) CNT-GO/NR composites.

The crack propagation paths of NR composites under the same tearing energy are shown in Figure 8. Compared with that of the pure NR matrix, the CNT/NR and GO/NR composites had more complex extension paths. This is because the CNTs and GO became stretched and oriented under external force at the crack tip, which effectively transmitted stress and improved the SIC performance of the NR. As illustrated in Figure 9a, GO with a large aspect ratio contributed to hindering the crack propagation. When the cracks encountered GO, the crack tips tended to avoid them and then continued to propagate.

After the addition of GO and CNT into the NR composites, the crack propagation paths became more complex; they exhibited passivation, branching, and deflection. The branched cracks of the CNT-GO/NR composites appeared earlier, and the angles between the main and the branched cracks were larger when compared with those of the GO/NR composites; this indicated that the CNT-GO network was more effective than the GO network in hindering crack propagation. The complicated paths were beneficial in reducing the crack growth rate. As illustrated in Figure 9b, fibrous CNTs and lamellar GO formed a filler network that comprised a fishing net-like structure, which effectively distributed stress and relieved stress concentration. When they encountered large CNTs or GO, the crack tips tended to avoid them and propagated toward a path with less resistance, thereby generating branched cracks. This process dispersed the energy of the main cracks, thereby reducing the crack growth rate and prolonging the fatigue life of the composite.

## 4. Conclusions

NR composites were fabricated with CNTs and GO to induce the formation of complex filler networks and enhance the fatigue properties of the composites. The TEM images of the CNT-GO/NR composites showed that the fibrous CNTs and the sheet-like GO were well dispersed and interpenetrated. Amongst the studied NR composites, the CNT-GO/NR composite demonstrated the strongest filler network and the highest storage modulus, tensile strength, and tear strength. The point of occurrence of the SIC in the CNT-GO/NR composites appeared earlier as compared to that in other composites. Owing to its complex filler network and the presence of SIC, the CNT-GO/NR composite exhibited the longest fatigue life, smallest crack precursor size, lowest crack growth rate, and more branching and deflections around crack tips, as compared to those of the other NR composites. In the engineering field, the NR composites filled with CNTs and GO could be used not only in tires and belts, but also in potential fields such as gas separation and evaporation. In addition, constructing chemical interfacial interactions between GO or CNTs with rubber macromolecules is recommended in the future research to further consume more tear energy and extend fatigue lives.

## Figures and Tables

**Table 1:** Composition of NR (natural rubber) filled with GO (graphene oxide) and CNTs (carbon nanotubes), phr ^a.^

Samples	NR	CNT/NR	GO/NR	CNT-GO/NR
Natural rubber	100	100	100	100
Zinc oxide	5	5	5	5
Stearic acid	1	1	1	1
Antioxidant RD ^b^	2	2	2	2
Antioxidant 4010 NA ^c^	2	2	2	2
Graphene oxide	-	-	2	1
Carbon nanotube	-	1	-	1
Accelerator DTDM ^d^	3.75	3.75	3.75	3.75
Accelerator DM ^e^	1.25	1.25	1.25	1.25
Sulfur	1.2	1.2	1.2	1.2

^a^ phr: parts by weight per hundred parts of rubber, ^b^ Poly (1,2-dihydro-2,2,4-trimethyl-quinoline), ^c^ N-Isopropyl-N’-phenyl-4-phenylenediamin, ^d^ 4,4’-Dithiodimorpholine, ^e^ 2,2’-Dibenzothiazoledisulfde.

**Table 2:** Absolute value of ∆G′ of NR composites with GO and CNT.

Sample	∆G′
CNT/NR	8.33
GO/NR	11.1
CNT-GO/NR	17.3

**Table 3:** Mechanical properties of NR composites.

Sample	NR	CNT/NR	GO/NR	CNT-GO/NR
Tensile strength (MPa)	22.4 ± 0.6	25.1 ± 0.7	28.7 ± 0.5	29.3 ± 0.8
Elongation at break (%)	495 ± 9	524 ± 14	515 ± 17	560 ± 16
100% Modulus (MPa)	1.2 ± 0.1	1.3 ± 0.1	1.6 ± 0.2	1.8 ± 0.1
300% Modulus (MPa)	4.3 ± 0.2	4.2 ±0.3	5.8 ± 0.3	6.4 ± 0.2
Hardness (shore A)	46 ± 1	49 ± 1	51 ± 1	52 ± 1
Tear strength (kN/m)	30 ± 0.3	34 ± 0.2	35 ± 0.4	37 ± 0.3

**Table 4:** *C*_1_ (intercept), *C*_2_ (slope), and *λ_up_* (the turning point of SIC occurrence) values obtained from the modified Mooney–Rivlin plots.

Sample.	*C*_1_ (MPa)	*C*_2_ (MPa)	*λ_up_*
NR	0.57	1.70	4.79
CNT/NR	0.90	1.38	3.98
GO/NR	1.66	1.81	3.51
CNT-GO/NR	1.51	0.76	2.87

**Table 5:** Average crack precursor sizes of the NR composites.

Sample	NR	CNT/NR	GO/NR	CNT-GO/NR
*c*_0_^1^ (μm)	218.1	206.0	192.4	185.1

^1^*c*_0_: crack precursor size.

## Supplementary Materials

The following are available online at https://www.mdpi.com/1996-1944/13/24/5746/s1, Table S1. Fatigue lives of NR composites under different strains, Table S2. Crack propagation rate of NR composites under different tearing energies.
