# Supplementary material for: Enhanced Fatigue and Durability Properties of Natural Rubber Composites Reinforced with Carbon Nanotubes and Graphene Oxide
Source: Materials (Basel). 2020 Dec 16;13(24):5746. doi: 10.3390/ma13245746 (PMC7767227; doi:10.3390/ma13245746)
Supplement: Supplementary file 1 [file materials-13-05746-s001.pdf]

materials

Supplementary Materials

Enhanced Fatigue and Durability Properties of Natural Rubber Composites Reinforced with Carbon Nanotubes and Graphene Oxide

Hao Guo [1,2], Peizhi Ji [1,2], István Zoltán Halász [3], Dávid Zoltán Pirityi [3], Tamás Bárány [3], Zongchao Xu [1,2], Long Zheng [1,2], Liqun Zhang [1,2], Li Liu [1,2,]* and Shipeng Wen [1,2,]*

[1] State Key Laboratory of Chemical Resource Engineering, Beijing University of Chemical Technology, Beijing 100029, China; 2018200559@mail.buct.edu.cn (H.G.); 2018210263@mail.buct.edu.cn (P.J.); 2020700020@mail.buct.edu.cn (Z.X.); 2017400089@mail.buct.edu.cn (L.Z.); zhanglq@mail.buct.edu.cn (L.Z.)
[2] Beijing Engineering Research Center of Advanced Elastomers, Beijing University of Chemical Technology, Beijing 100029, China
[3] Department of Polymer Engineering, Faculty of Mechanical Engineering, Budapest University of Technology and Economics, Műegyetem rkp. 3., H-1111 Budapest, Hungary; halaszi@pt.bme.hu (I.Z.H.); pirityid@pt.bme.hu (D.Z.P.); barany@pt.bme.hu (T.B.)
* Correspondence: liul@mail.buct.edu.cn (L.L.); wensp@mail.buct.edu.cn (S.W.); Tel.: +86-010-64434860

Table S1. Fatigue lives of NR composites under different strains.

Strain (%)	NR	CNT/NR	GO/NR	CNT-GO/NR
100	49835 ± 1336	68314 ± 1235	85324 ± 3313	89146 ± 2256
150	3018 ± 932	37489 ± 1098	40315 ± 1341	43092 ± 2301
200	9379 ± 857	14772 ± 1853	14822 ± 1072	17113 ± 1892
250	193 ± 53	606 ± 179	1739 ± 281	2083 ± 201
300	84 ± 41	198 ± 65	475 ± 67	582 ± 83

Table S2. Crack propagation rate of NR composites under different tearing energies.

T (J/m²)	NR	CNT/NR	GO/NR	CNT-GO/NR
300	14.74 ± 1.11	8.28 ± 0.98	6.11 ± 0.63	5.84 ± 0.65
500	16.17 ± 1.26	10.75 ± 1.02	6.98 ± 0.88	6.85 ± 0.76
1000	19.76 ± 1.58	13.22 ± 1.31	12.55 ± 1.27	8.39 ± 0.92
1500	45.13 ± 2.13	16.78 ± 1.55	15.91 ± 1.46	12.9 ± 1.04
1800	86.52 ± 3.32	68.71 ± 2.01	47.53 ± 2.22	33.04 ± 2.11

Publisher's Note: MDPI stays neutral with regard to jurisdictional claims in published maps and institutional affiliations.

© 2020 by the authors. Submitted for possible open access publication under the terms and conditions of the Creative Commons Attribution (CC BY) license (http://creativecommons.org/licenses/by/4.0/).
